# Endurance Exercise Training Alters Lipidomic Profiles of Plasma and Eight Tissues in Rats: a MoTrPAC study

**DOI:** 10.21203/rs.3.rs-5263273/v1

**Published:** 2024-11-21

**Authors:** Eric Ortlund, Zhenxin Hou, Chih-Yu Chen, David Gaul, Tiantian Zhang, Samuel Moore, Xueyun Liu, Anna Ivanova, Kristal Maner-Smith, Christopher Newgard, Sue Bodine, Evan Savage, Alexis Bennett, Facundo Fernandez

**Affiliations:** Emory University School of Medicine; Emory University; Emory University; Georgia Tech; Emory University; Georgia Institute of Technology; Emory University; Centers for Disease Control and Prevention (CDC); Emory University School of Medicine; Duke University School of Medicine; Oklahoma Medical Research Foundation; Georgia Institute of Technology; Georgia Institute of Technology; Georgia Institute of Technology

**Keywords:** MoTrPAC, Endurance Exercise Training (ExT), lipidomics, sexual dimorphism, multi-tissue

## Abstract

Endurance exercise training (ExT) induces metabolic, structural, and functional adaptations via lipidomic modifications, yet the systematic elucidation of lipidome alterations in response to ExT remains incomplete. As a part of the Molecular Transducers of Physical Activity Consortium (MoTrPAC), we leveraged non-targeted and targeted lipidomics for the systematic discovery of lipid alterations in the brown adipose tissue, heart, hippocampus, kidney, liver, lung, skeletal muscle gastrocnemius, subcutaneous white adipose tissue, and plasma in response to 1, 2, 4 or 8 weeks of ExT in 6-month-old male and female Fischer-344 rats. This study demonstrates that these tissues, each with distinct lipidomic features, underwent dynamic, sexually dimorphic lipid remodeling. Exercise trained animals showed reduced whole-body adiposity and improved cardiorespiratory fitness, along with enhanced utilization of lipid stores and dynamic triacylglycerol remodeling compared to sedentary controls in all tissues except hippocampus. They also showed modifications in phospholipids, lysophospholipids, oxylipins, and ceramides in several tissues. Coordinated changes across tissues reflect systemic tissue communication, with liver-plasma-heart connection potentially playing a key role in systemic lipid metabolism during ExT. These data will improve our understanding of lipid-associated biological processes underlying the health-promoting benefits of ExT.

## Introduction

Lipids are critical biological effectors, functioning as metabolic substrates, signaling molecules, and cellular membrane components^1^. Abnormal lipid accumulation and metabolism in tissues, such as liver and skeletal muscle, has been associated with a range of disease risks and metabolic disorders, including obesity, insulin resistance, type 2 diabetes (T2D), cardiovascular disease, and non-alcoholic fatty liver disease (NAFLD)^2–5^. For example, excessive accumulation of triacylglycerols (TAGs), stemming from dysregulation of lipid homeostasis, is a hallmark of obesity and NAFLD^5^. Phospholipids and sphingolipids are implicated in mediating insulin action, influencing mitochondrial dynamics, and modulating gene expression^2,3^. Hence, lipid metabolic pathways are potential therapeutic targets to counter metabolic disease onset and progression^2^.

Exercise confers numerous health benefits. Endurance exercise training (ExT) alters metabolism in oxidative tissues and induces structural and functional adaptations^6,7^. Nonetheless, the impact of exercise on systemic modifications in lipid metabolism and flux is not fully understood. Inconsistencies persist in the literature regarding how exercise regulates tissue lipid levels, such as intrahepatic triglycerides (IHTG) and intramuscular triglycerides (IMTG)^5,8^, and how these modifications contribute to metabolic health. Past research has identified an inverse relationship between IMTG content and insulin sensitivity, but athletes exhibit comparable levels of IMTG as diabetic patients, while being extremely insulin sensitive^8^. This is known as the ‘athlete’s paradox’ and has led to various hypotheses regarding the excess accumulation of lipid intermediates, such as diacylglycerols (DAG), ceramides, long-chain acyl CoAs, and acylcarnitines as the culprit; however, results from studies focusing on these lipids in exercise have also been inconsistent^8^. In addition, exercise induces lipidomic responses across various tissues, but most previous studies have focused on changes in a single tissue, such as skeletal muscle, liver, adipose tissues, or plasma/serum. By understanding how exercise modulates lipid remodeling in multiple tissues and cross-tissue connectivity, we expect to gain deeper insights into the roles of lipids in mediating metabolic diseases, paving the way for developing new therapeutic targets.

To begin to fill these knowledge gaps and provide a comprehensive resource for multi-tissue lipidomic responses to ExT, as a part of Molecular Transducers of Physical Activity Consortium (MoTrPAC), we applied non-targeted high-resolution lipidomics, complemented by sensitive targeted lipidomic assays to deeply characterize the lipidomes of male and female Fischer 344 rats subjected to 1, 2, 4, and 8 weeks of ExT. We identified and quantified thousands of lipids and lipid mediators in eight tissues and plasma. We examined the sex-specific temporal lipidomic changes in response to ExT, and subsequently tested whether lipid abundance changes were associated with sexually distinct changes in phenotypic characteristics. We further examined changes in the abundances and compositions of glycerolipids, phospholipids, sphingolipids, and lipid mediators. Finally, we explored the tissue connectivity during the 8-week ExT. We generated a comprehensive multi-tissue lipidome map of ExT response in rats, providing a valuable data resource for future investigations of exercise metabolism and for the identification of lipid transducers that mediate exercise adaptations and the beneficial effects of exercise training.

## Results

### Lipidome profiling of eight tissues and plasma

**Figure 1:**
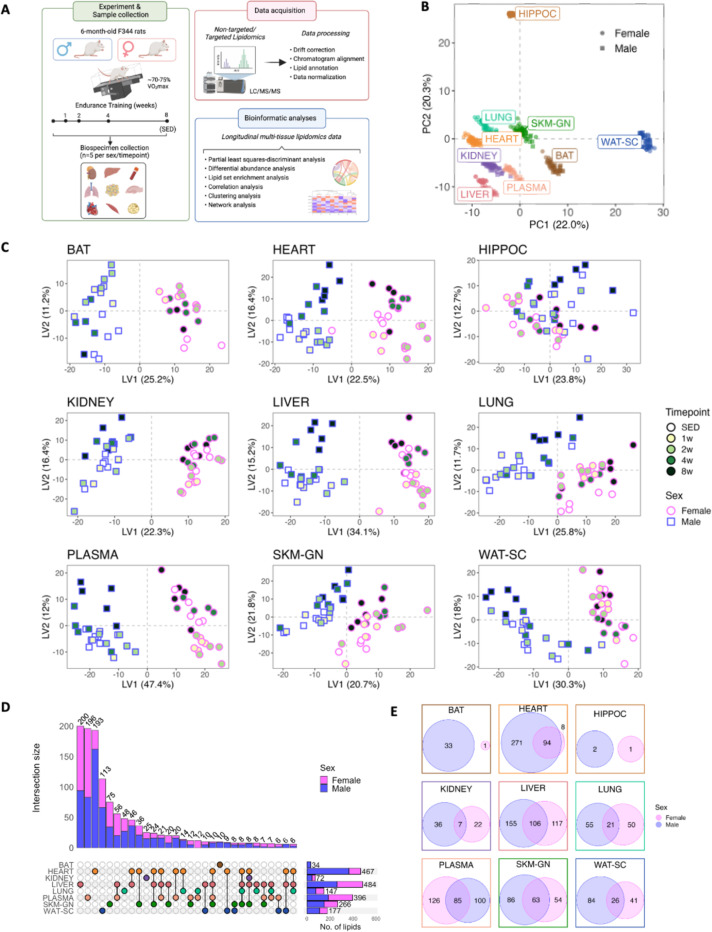
Lipidomic profiling of eight tissues and plasma (A) Study design. Eight tissues and plasma samples were collected from 6-month-old male and female Fischer-344 rats that were trained for 1, 2, 4, or 8 weeks (n=5) 48 hrs post last exercise bout for lipidomic analyses. (B) Principal component analysis (PCA) of tissue lipidomes, based on sample-level normalized data of 505 lipids common to all 8 tissues and plasma, i.e., lipids with >50% representation (detected in ≥5 tissues/plasma). The remaining missing values were replaced with the minimum level of each lipid. Color and shape indicate tissue and sex, respectively. (C) Partial least squares-discriminant analysis (PLS-DA) plots of tissue lipidomes. Color and shape indicate training group and sex, respectively. (D) Upset plot of number of lipids that responded to exercise training (FDR < 0.05). (E) Venn diagram of number of lipids that responded to exercise training (FDR < 0.05).

Six-month-old, male and female Fischer 344 rats were exercised on a motorized treadmill 5 days per week using a progressive training protocol at a targeted intensity of 70–75% VO_2_max or remained sedentary (SED) for 8 weeks (Fig. 1A). After 1, 2, 4, or 8 weeks of ExT, eight tissues and plasma were collected (n = 5 per sex per timepoint) 48 hours after the last training session for lipidomic analysis. Using non-targeted lipidomics, we detected and quantified between 7,356 − 13,780 lipids and metabolites in each tissue, including 524–751 annotated lipids in each tissue that represent 43 lipid classes (Fig S1A, S1B). We also conducted targeted lipidomics assays that detected and quantified between 9–31 oxylipins and N-acylethanolamines (NAE) in each tissue (Fig S1A). These are oxidized derivatives of polyunsaturated fatty acids (PUFA) and function as lipid mediators for various physiological processes.

Principal component analysis (PCA) revealed that lipidomic variations across tissues greatly exceeded those between sexes or individuals. Variance component analysis estimated that the tissue contribution to the total variability in each of the first four principal components were all over 98% (99.0%, 98.9%, 99.3%, 99.4% for principal components 1–4, respectively). Lipid compositions of WAT-SC and HIPPOC were particularly unique compared to other tissues (Fig. 1B). We then performed semi-absolute quantification of lipids by normalizing peak areas using ISTDs and converted to concentrations. TAG made up > 90% of the total lipid concentration in WAT-SC and BAT (Fig S1D). Phosphatidylcholine (PC) represented the second most diverse lipid class, with 316 PC species detected across all tissues (Fig S1B). PC was the most abundant lipid class in 4 tissues (heart, liver, lung, SKM-GN; Fig S1D). The HIPPOC contained 178 unique lipids that were not detected in other tissues (Fig S1C) and was uniquely enriched in hexosylceramides (HexCer), accounting for 13.1% of total lipids (Fig S1B, S1D). Ether lipids (e.g., plasmalogens) are known to be enriched in the brain and heart^9^. We observed that ether phosphatidylethanolamines (O-PE) represented the most abundant lipid class in HIPPOC (33.5%) and the third in heart (12.6%), being more abundant than phosphatidylethanolamine (PE) in HIPPOC, lung, plasma, and WAT-SC (Fig S1B, S1D). Interestingly, there were also 117 lipids that were only detected in plasma, including 47 lysophosphatidylcholine (LPC) and 7 lysophosphatidylethanolamine (LPE) species (Fig S1C).

Partial least squares-discriminant analysis (PLS-DA) revealed that variations in tissue-specific lipidomic profiles were mainly accounted for by sex (Fig. 1C). ANOVA-estimation for variance components estimated that sex contributed to 75–96% of total variability in the first latent variable for all tissues and plasma except for HIPPOC, highlighting profound sexual dimorphism in tissue lipidomes (Fig. 1C). Several metabolically active tissues, such as heart, liver, SKM-GN, and WAT-SC, exhibited modified lipidomic profiles after 8 weeks of ExT, particularly in males (Fig. 1C). We then performed differential analysis comparing trained animals (across all timepoints) to sedentary controls separately for males and females for each tissue, given the profound sex differences. Over 50% of annotated lipids (916/1716) showed differential abundances during ExT in at least one sex or tissue (FDR < 5%). Overall, ExT-induced lipidomic changes were tissue dependent, as significantly altered lipids were mostly tissue-specific (Fig. 1D). Within each tissue, male and female rats also showed distinct responses, as indicated by the low numbers of shared significantly altered lipids (Fig. 1E). More than half of the lipids (58%) responded to exercise training in the male heart, whereas female heart showed a much lower response (16%; Fig. 1D, 1E), making heart one of the most sexually dimorphic tissues in ExT response. Liver and plasma also showed robust ExT responses, with approximately a third of total lipids altered during 8-week ExT (Fig. 1D, 1E).

### Sexual dimorphism in tissue lipidomes

**Figure 2:**
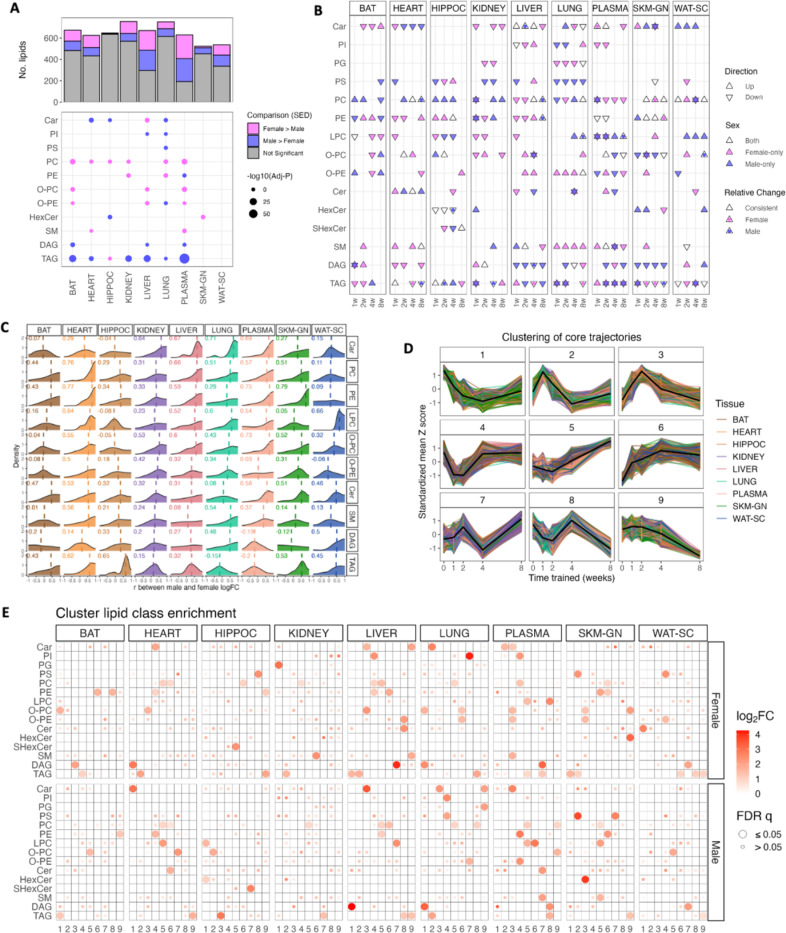
Sex-specific temporal *ExT* responses in lipidomes (A) Number of lipids and enrichment plot of lipid classes that were significantly different between sexes at SED (FDR < 0.05). Color and dot size indicate sex comparison and enrichment significance, respectively. (B) Enrichment plot of lipid classes that responded to exercise training at each training timepoint (FDR < 0.05). Direction of the triangle indicates the sign of normalized enrichment scores (NES) as determined by LSEA. When males and females both show significant changes in the same direction, a colored dot inside the white triangle is used to indicate which sex exhibits significantly stronger changes. (C) Density plot of correlation coefficients between male versus female lipid timewise logFC, showing tissue as columns and lipid classes as rows. Dashed line and number indicate median. (D) Clustering of longitudinal trajectories of lipids. Line plots show the standardized group means (n = 5) of sample-level values of lipids in the cluster cores (membership values > 0.5) in identified clusters. (E) Over-represented lipid classes in each cluster. Heatmap shows logFC as dot color and enrichment adjusted p-value as dot size, as determined by the overrepresentation analysis (ORA) based on one-sided Fisher exact t tests.

To elucidate sex differences tissue lipidomes, we directly compared lipid abundances by performing differential analysis between sexes at baseline (i.e., SED group, Fig. 2A) and at individual ExT timepoints (Fig S2A). Consistent with PLS-DA results in Fig. 1C, robust sex differences existed in several tissues and were largely maintained throughout ExT (Fig S2A). In the plasma, 445 (69.5%) lipids were significantly different between sexes at baseline, with 279 lipids different throughout all timepoints (Fig. 2A, S2A). In the liver, 325 (55.8%) were significantly different, with 169 different throughout (Fig. 2A, S2A). Moderate sex differences were also present in BAT, heart, kidney, lung, and WAT-SC (~ 20–30% lipids significantly different; Fig. 2A, S2A). Rank-based lipid class enrichment analysis reveal that male tissues were more enriched in glycerolipids (TAG and DAG), whereas female tissues were more enriched in phospholipids (glycerol- and ether-phospholipids) (Fig. 2A).

### Temporal ExT responses in tissue lipidomes

To understand ExT effects on tissue lipidomes, we sought to identify differentially abundant features at each training timepoint compared to sedentary animals (SED) within each sex. Overall, females showed stronger responses at early timepoints (1w, 2w), whereas males tended to show delayed responses to ExT, with the number of altered lipids in male heart, liver, kidney, SKM-GN, WAT-SC, and plasma increasing drastically at 8w (Fig S2B). Notably, SKM-GN of both male and female rats showed steadily increased number of significantly altered lipids with time (Fig S2B). Interestingly, the female lung displayed a greater number of significantly altered lipids at all timepoints than males (Fig S2B).

With the differential analysis results, we performed Lipid Set Enrichment Analysis (LSEA), a rank-based enrichment analysis, to evaluate temporal ExT responses of 15 lipid classes. Results reveal strong changes in many lipid classes including glycerolipids, phospholipids, and sphingolipids. Among them, TAG, PC, ether phosphatidylcholine (O-PC), and LPC showed significant enrichment in all tissues, followed by DAG, sphingomyelin (SM), O-PE, and acylcarnitines (Car) in 8 tissues, although the strength and direction of enrichment varied by tissue, sex, and timepoint (Fig. 2B). TAG showed positive enrichment at 1–2w before negative enrichment at 8w (e.g., heart, liver, lung, SKM-GN). PC and PE showed similar temporal ExT responses, with several tissues showing overall positive enrichment, including SKM-GN, HIPPOC, liver, heart (at later timepoints in females), and WAT-SC (PC only). For DAG, male liver and SKM-GN both showed significant negative enrichments throughout ExT, whereas reduction in DAG occurred in the female heart. Lung and plasma DAGs in both sexes showed moderate and delayed reduction starting at 2w. Hepatic and SKM-GN DAGs are clinical markers for the development of insulin resistance^1,10,11^, and increased DAG has been reported in NAFLD patients^12,13^. Reduction of DAGs in both SKM and liver DAGs in male rats may suggest improvement in insulin sensitivity.

For lipids that showed sex-consistent changes (i.e., males and females both showed significant changes in the same direction; indicated by a white triangle in Fig. 2B), we further tested for sex differences in the magnitude of temporal ExT responses between sexes by running a differential analysis contrasting sex-specific ExT response in each time-matched group (e.g., (male 1w – male SED) – (female 1w – female SED)). A colored dot within the white triangle is used to indicate which sex exhibits significantly stronger changes. For example, while both sexes showed positive enrichment in PC and PE in liver and SKM-GN at later time points, the increases in male liver were stronger than female at 8w, whereas increases in female SKM-GN were stronger at 4w and 8w (Fig. 2B). TAG showed highly sex-specific responses in liver, BAT, and plasma.

To further compare ExT effects on lipids between sexes, we calculated Pearson correlations between male and female time-resolved logFC for lipids within each tissue and visualized correlation coefficient distributions. Car, PC, and PE mostly showed a distribution with a dense positive correlation, suggesting relatively consistent training responses between sexes, whereas SM, DAG, and TAG showed weaker, albeit still positive correlations, suggesting relatively sexually dimorphic post-ExT changes (Fig. 2C). On the tissue level, heart, SKM-GN and liver showed moderately sex-consistent changes in lipid abundance with ExT, with tissue-level median correlation coefficients above 0.45. Whereas other tissues exhibited lower median correlation coefficients of around 0.3 (Fig. 2C), suggesting sex-specific temporal lipidomic ExT responses in most tissues. Interestingly, previous analyses (Fig. 1E, S2B) showed different numbers of significantly altered lipids in female versus male heart. To further explore sexual dimorphism in the heart, we examined sex-specific changes in heart lipid abundances. Out of the 271 male-specific training-altered lipids in Fig. 1E, 84 lipids were significantly correlated between male and female time-resolved logFC (trajectories of top 20 of these 84 lipids were visualized in Fig S2C). Together, this suggests that female and male hearts showed similar directions of post-training changes, but greater magnitude of change in males, causing more lipids to reach a statistical significance cutoff of 5% FDR (Fig. 1E, S2B).

Using c-means clustering, nine main clusters of longitudinal trajectories were identified that characterized different temporal patterns during ExT (Fig. 2D). We then performed over-representation analysis (ORA) to characterize each cluster (Fig. 2E). Plasma, lung, and liver acylcarnitines (mainly medium- and long-chain) were enriched in clusters 2 and 3, which showed an early increase and later returned towards baseline. This was opposite to heart acylcarnitines, which were enriched in clusters 1 and 4 that were characterized by an early decrease. PC and PE were enriched in clusters 4, 5, and 6 for heart, HIPPOC, kidney, liver, lung, plasma, and SKM-GN in both females and males, and were mainly characterized by persistent increases (cluster 6) or a delayed increase (cluster 5). Consistent with previous observations, male liver and lung DAGs were enriched in cluster 1, decreasing at early timepoints and maintained low levels. TAGs were enriched in cluster 9 in male heart, liver, SKM-GN, and WAT-SC, exhibiting a drastic decrease with prolonged ExT. On the other hand, for females, liver and SKM-GN TAGs were enriched in clusters 1 and 2, and heart and kidney TAGs were enriched in cluster 2, indicating divergent early responses before reduction with prolonged ExT. Female WAT-SC TAGs were spread in clusters 6, 8, and 9 (the majority of species in cluster 8), indicating complex and dynamic modifications of the female WAT-SC TAG pool. Together, these analyses reveal sex-specific, dynamic utilization and adaptation of lipids across multiple tissues during the 8-week ExT.

### Integrative phenotypic-lipidomic responses to ExT

**Figure 3:**
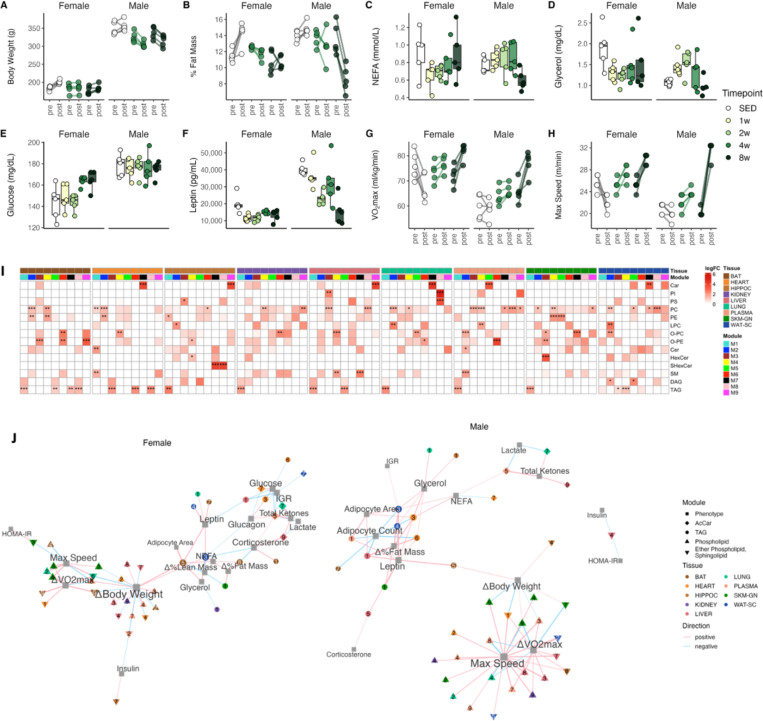
Integrative phenotypic-lipidomic responses to ExT (A-H) Measurements of selected phenotypic traits: (A) body weight, (B) %fat mass, circulating plasma levels of (C) NEFA, (D) glycerol, (E) glucose, (F) leptin, (G) VO_2_max, and (H) max speed. (I) Over-represented lipid classes in each tissue module. Heatmap shows logFC as dot color and asterisks indicate statistical significance (*, p<0.05; **, p<0.01; ***, p<0.001), as determined by the ORA based on one-sided Fisher exact t tests. (J) WGCNA network of selected phenotype-module correlations. Line colors indicate signs of significant correlations (p-value < 0.05; red, positive; blue, negative). Node and font size indicate degree of the node (number of its adjacent edges). Each node is labeled with module number or phenotype, and the shape of node indicates the significantly enriched lipid class.

Eight-week ExT led to robust, sex-specific phenotypic and physiological changes, which were described in detail in related studies (Fig. 3A-H, S3A-H)^14–16^. To link lipidomic modifications to phenotypic changes, we used weighted gene co-expression network analysis (WGCNA) to cluster lipids based on their profiles. Hub lipids (membership ≥ 0.7; Fig S3I) were used in ORA to characterize each module. Results of module membership and ORA are presented in Table S1, S2 and Fig. 3I, respectively. We then assessed correlations of tissue module eigenvalues (MEs), which can be considered a representative of the module profile, with selected clinical plasma analytes and phenotypic measures (Fig. 3J, Table S3).

Males exhibited weight loss and reduced fat mass, whereas females did not show decreases in body weight and fat mass, yet exercise prevented them from gaining weight as observed in control females (Fig. 3A, 3B). Both sexes showed increased lean mass (Fig S3A). As expected, many TAG modules (heart M3 and M6, liver M1, SKM-GN M1, WAT-SC M3 and M4) were positively correlated with %fat change in males (Fig. 3J). Correlations between male WAT-SC TAG (M3 and M4) modules and adipocyte area and adipocyte count, as described in more details by Many et al^14^, provided evidence for exercise-promoted lipolysis that changed adipocyte morphology (i.e., more abundant, smaller adipocytes), which were absent in females. Nevertheless, females TAG modules in SKM-GN (M1), WAT-SC (M3), and BAT (M5, M8) were positively correlated with body weight change, and/or %fat change, and negatively correlated with %lean change (Fig. 3J). These correlations provided evidence that exercise promotes fat loss and weight loss and reduced whole-body adiposity via stimulating lipolysis and promoting TAG catabolism in various tissues including WAT-SC, SKM-GN, BAT, liver, and heart^17,18^.

Circulating non-esterified fatty acids (NEFA) and glycerol increased initially but decreased at 8w in males (Fig. 3C, 3D), and were positively correlated with several TAG modules (heart M3 and M6, WAT-SC M3 and M4, lung M1, HIPPOC M1; Fig. 3J), the MEs of which showed similar temporal trends (Fig S3J). Circulating glycerol reflects whole-body lipolysis during fasting and mainly comes from stored TAG, but also from glucose and lactate^19,20^. These correlations support enhanced TAG mobilization following lipolysis from these tissues at early time points and diminished as a result of the depleted TAG store at 8w in males as reported previously^14^ (also discussed later). For females, circulating NEFA and glycerol decreased immediately after 1w, and remained low (Fig. 3C, 3D). Female BAT TAG (M5, M8) and WAT-SC TAG (M3) were positively correlated with circulating NEFA and/or glycerol. These three modules showed an initial reduction but somewhat recovered later. These associations likely reflect the turnover and preservation of TAG depots in female adipose tissues in response to prolonged ExT.

Several tissue modules that were overrepresented by acylcarnitines (lung M7, plasma M5, liver M9) consistently showed an initial increase and later returned towards baseline levels in both sexes (Fig S3J). These modules were positively correlated with circulating ketones and negatively correlated with lactate in both sexes (Fig. 3J, S3E, S3F). The positive correlations between circulating ketone bodies and tissue acylcarnitines reflect accelerated fatty acid β-oxidation (FAO) providing elevated levels of acyl-CoAs for biosynthesis of ketone bodies (using acetyl-CoA) and acylcarnitines (using medium- and long-chain acyl-CoAs). The later reduction in acylcarnitines and ketone bodies may reflect an improvement in oxidative capacity that diminished the accumulation of FAO products and thus acylcarnitine synthesis and ketogenesis. This effect could also be due to an elevated ability to utilize ketone bodies in exercise-trained oxidative tissues such as heart and skeletal muscle^21,22^.

We also explored hormonal regulation of energy metabolism during ExT. Circulating leptin decreased with exercise in both sexes (Fig. 3F; females initially, and males progressively), and was positively associated with multiple TAG modules in both sexes (Fig. 3J; male: BAT M5, heart M3 and M6, liver M1 and M5, plasma M1, SKM-GN M1, WAT-SC M3 and M4; female: BAT M5 and M7, lung M1, WAT-SC M3 and M4), supporting the notion that circulating levels of leptin are proportional to fat mass^23,24^. Males exhibited a lower insulin:glucagon ratio (IGR) at 8w due to a slight increase in circulating glucagon and a decrease in insulin (Fig S3B-S3D), consistent with previous studies that report ExT decreases insulin and increases glucagon secretion^22^. It is also consistent with enhanced mobilization and catabolism of stored lipids stimulated by a low IGR at 8w^25^. On the other hand, female glucagon decreased at 4w and 8w, leading to an elevated IGR (Fig S3B-S3D). Heart (M3) and liver (M1) TAG modules were positively correlated with glucagon and negatively correlated with IGR and glucose (Fig. 3J, Table S3). Elevated IGR, which works to stimulate fat preservation^25^, was consistent with the female preservation of TAG at later timepoints.

Both male and female rats showed substantial increases in VO_2_max and maximum run speed with 8-week ExT (Fig. 3G, 3H), indicating improvement in cardiorespiratory fitness. We found positive correlations between VO_2_max change and post-training maximum run speed with several tissue modules that were overrepresented by PC and/or PE (Fig. 3J; male: heart M2; kidney M4 and M9; liver M3 and M8; lung M2 and M4; plasma M3, M4, M7, and M9; SKM-GN M2, M4, M5; WAT-SC M2 and M5; female: heart M1, lung M2, SKM-GN M2, M4, and M5). Notably, the robust correlations between SKM-GN PC, PE (M2, M4, and M5) and post-training max speed implicated an association between phospholipid remodeling in SKM-GN and exercise performance (discussed later).

### ExT effects on lipids in energy metabolism

**Figure 4:**
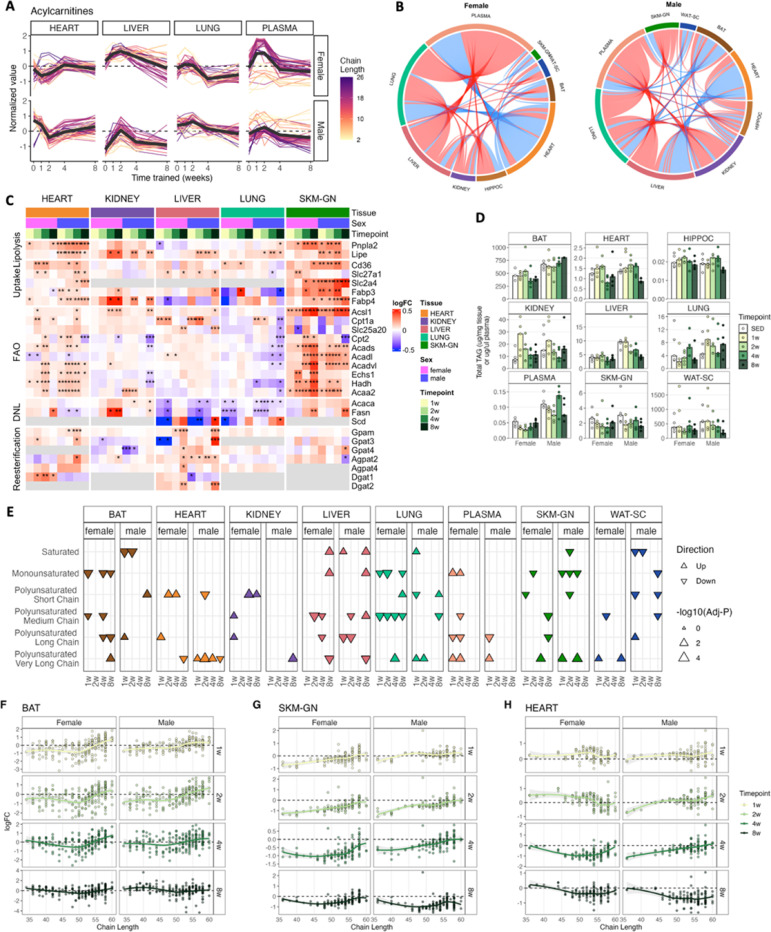
ExT effects on lipids in energy metabolism (A) Trajectory plots of standardized abundance of acylcarnitines in heart, liver, lung, and plasma. Colors indicate chain length. (B) Inter-tissue acylcarnitine logFC correlations. Correlations were performed using logFC trajectory of the same lipid between tissue pairs. Colored sectors indicate tissue. Ribbons indicate correlations between tissues, with colors indicating signs of correlation coefficients (red, positive; blue, negative). Only |r| > 0.8 were displayed. (C) Heatmap showing fold changes for selected proteins that are involved in fatty acid metabolism and TAG remodeling (*, p<0.05; **, p<0.01; ***, p<0.001). (D) Total TAG content. (E) Enrichment plots of TAG of different chain lengths and double bonds, grouped as saturated, monounsaturated, and polyunsaturated species, which were further categorized as short-chain (C30–48), medium-chain (C49–52), long-chain (C53–55), and very-long-chain (C56–60). (F-H) Timewise logFC versus the number of total carbons of TAG in (F) BAT, (G) SKM-GN, (H) heart. Row and column indicate total carbon and double bond number.

As described above, acylcarnitines showed coordinated increases of median- and long-chain acylcarnitines (≥ C12) in lung, liver, and plasma during early timepoints, which has been widely reported in human plasma with ExT^26–28^. On the other hand, heart showed opposite temporal changes (Fig. 4A), leading to positive correlations across lung, liver, and plasma, and negative correlations with heart (Fig. 4B). Elevated hepatic acylcarnitines (≥ C12), paired with elevated levels of acetyl-carnitine, reduced levels of acetyl-CoA, and increased Cpt1a protein abundance (Fig. 4C, S4A), may reflect partial FAO when influx of long-chain fatty acyl CoA into the mitochondria exceeds the capacity for FAO and subsequent TCA cycle^26–28^. This effect was likely due to elevated lipolysis in oxidative tissues and TAG-storing adipose tissues, resulting in an excess of acylcarnitines entering circulation from tissues. On the other hand, the depletion of almost all acylcarnitines (C2-C22) in heart at early timepoints and elevated levels of free carnitine (Fig. 4A, S4B) may suggest an increased utilization of fatty acids for oxidation in the heart that exceeds its supply, limited by acyl CoA shuttling into mitochondria or the rate of lipolysis rather than carnitine deficiency in the early response to ExT. Heart showed greater increased abundances of β-oxidation proteins (Acads, Acadl, Acadvl, Echs1, Hadh, Acaa2) than Acsl1 and Cpt1, key proteins involved in fatty acid shuttling into mitochondria, whereas the liver and lung showed stronger up-regulation of Cpt1 (except female lung), with no significant changes in β-oxidation proteins (Fig. 4C). This provides more evidence for different adaptations to enhanced energy requirements by the heart compared to the liver. Notably, plasma short-chain acylcarnitines (acetyl-carnitine, C3-C6, C10) were higher in males than females, while the opposite was true for median- and long-chain acylcarnitines (≥ C12; Fig. 4A). Odd-chain (C3, C5) acylcarnitines are catabolic products of branched chain amino acids. Therefore, this may reflect sex differences in the utilization efficiency of substrates. That is, males are more capable of utilizing fatty acids whereas females are more capable of utilizing branched chain amino acids for FAO. Additionally, male heart, liver and plasma Car 24:0, Car 24:1, Car 26:1 showed significant increases at early timepoints of ExT (Fig S4C). This may suggest an overload of peroxisome of long-chain and very-long-chain fatty acids for β-oxidation, as peroxisomes contribute to roughly 15–45% of the oxidation of C16–24 fatty acids^29^. At 8w, acylcarnitine levels returned towards baseline in both sexes (Fig. 4A), likely reflecting adaptive responses in elevated FAO capacity due to increased mitochondrial oxidative capacity^30^.

TAG represented the most diverse lipid class, with 404 molecular species detected across all tissues (Fig S1B). Consistent with previous results (Fig. 2A), higher levels of TAG were present in males than females at baseline in BAT, kidney, liver, lung, plasma, and WAT-SC (Fig. 4D). During the 8-week ExT, SKM-GN showed immediate reduction of total TAG content after 1w, whereas other tissues (heart, male lung, female kidney) showed delayed reduction, or even an initial increase, which may suggest preferential flux to these tissues in response to elevated energetic demands from ExT. Male WAT-SC, liver, SKM-GN, and heart TAG content decreased drastically (~ 70%, ~ 55%, ~ 50%, and ~ 35% respectively) after 8 weeks of ExT, whereas females showed rebound of TAG content in these tissues after 8w (relative to 2w or 4w; Fig. 4D). Heart and SKM-GN showed increased Cd36, Fatp1, Fabp3, Fabp4 protein abundance, particularly in males, reflective of active uptake of fatty acids. Liver and SKM-GN DNL protein abundances (Acaca, Fasn, Scd) decreased at 1–2w, but recovered or surpassed the SED level in 4w and 8w in both sexes (Fig. 4C). The drastic increase in Acaca protein abundance was noted in female WAT-SC at 8w^14^. Fasn increased in female heart at 8w but decreased in male heart. In addition, lipolytic activities (ATGL, HSL) increased in heart, SKM-GN, liver, and WAT-SC, and were stronger in male than female heart. Together, these data show ExT enhances lipid utilization and suppresses lipid synthesis at early exercise time points, accelerating TAG utilization and therefore fat loss.

Concentrations of individual TAG species exhibited a wide range – over five orders of magnitude in each tissue (except HIPPOC; *data not shown*). Given the complexity of the tissue TAG pool, their dynamic responses to ExT were unraveled by examining the differential responses of TAG with different chain lengths and unsaturation levels. Overall, many tissues (e.g., SKM-GN, BAT, WAT-SC) showed greater reduction of saturated and monounsaturated, and short- and medium-chain (C36–50) polyunsaturated TAG species than very-long-chain (C56–60) polyunsaturated species. The latter either increased relative to SED or decreased at a slower rate than other TAG species (Fig. 4E-G, S4D, S4E and by Many et al.^14^), especially at early timepoints in both sexes. This could reflect preferential utilization of more saturated and shorter-chain TAG species. In heart, males exhibited similar preservation of very-long-chain TAG species at the expense of short-chain TAG before 8w, whereas females preferentially preserved short-chain TAG species (C36–44) (Fig. 4H, S4F). Consistent with this observation, female heart exhibited increases in Dgat1 throughout ExT whereas male Dgat1 levels decreased, suggesting an active recycling of TAG in female heart (Fig. 4C). In the liver, females exhibited preservation or increases in selected TAG species at 1w, 2w and 8w, whereas males showed reduced TAG concentrations starting at 2w (Fig. 4D, S4G). Consistently, we observed female-specific increases in Gpat4 and Agpat4, in addition to increased protein abundances of Gpam, Gpat3, Dgat2 in both sexes at 8w (Fig. 4C). Together, these data suggest ExT-induced dynamic TAG remodeling via enhanced lipid synthesis and turnover with 8 weeks of ExT, potentially driving the TAG rebound to compensate for the loss of TAG store in females.

### ExT effects on phospholipids

**Figure 5:**
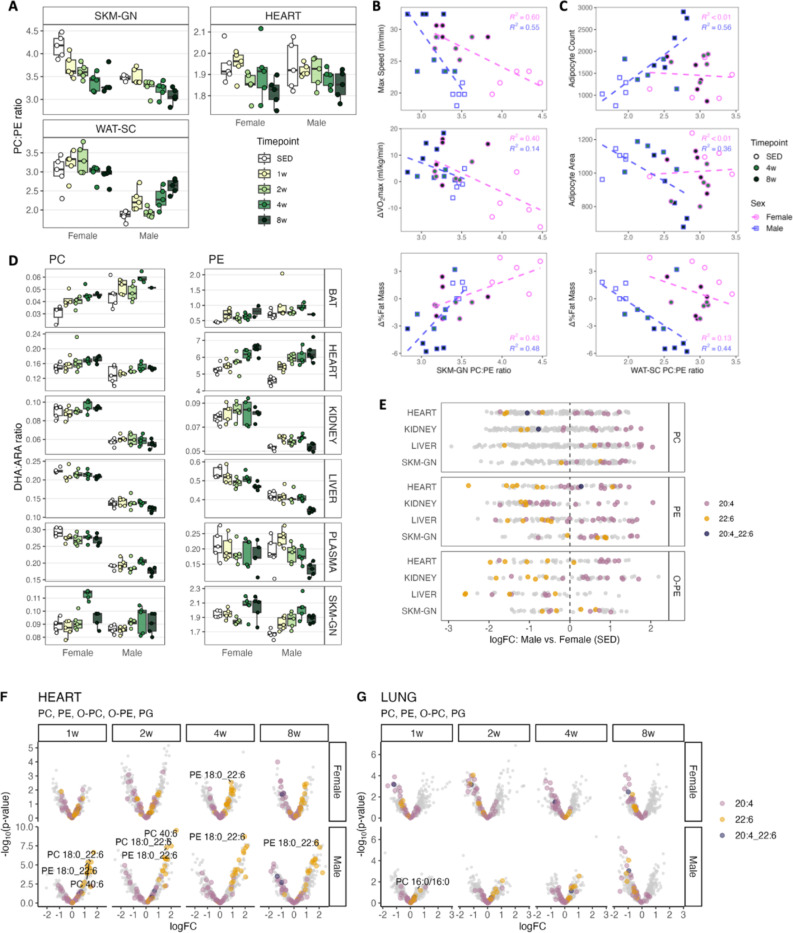
ExT effects on phospholipids (A) PC:PE ratios of SKM-GN, heart, and WAT-SC. (B) Correlations between SKM-GN PC:PE ratio and max speed, VO_2_max change, %fat change. (C) Correlations between WAT-SC PC:PE ratio and adipocyte count, adipocyte area, %fat change. (D) PC and PE DHA:ARA ratios of BAT, heart, kidney, liver, plasma, and SKM-GN. (E) Scatterplots displaying comparisons of phospholipid (PC, PE, O-PE) abundances between male and female sedentary controls (logFC>0 indicates higher levels in males). Colors highlight phospholipids that contain 20:4 and/or 22:6 (grey: other phospholipids). (F) Volcano plots displaying comparisons of each trained group against sex-matched sedentary controls in heart. Colors highlight phospholipids (PC, PE, O-PC, O-PE, PG) that contain 20:4 and/or 22:6 (grey: all other lipids). (G) Volcano plots displaying comparisons of each trained group against sex-matched sedentary controls in lung. Colors highlight phospholipids (PC, PE, O-PC, PG) that contain 20:4 and/or 22:6 (grey: all other lipids).

Previous analyses revealed the overall increases of PC and PE – an immediate increase or an increase after an initial reduction – with ExT in heart, kidney, liver, lung, SKM-GN, and plasma (Fig. 2B, D, E; clusters 4–6). We calculated PC:PE ratios as they could reflect phospholipid composition of cellular membranes, and imbalances in PC:PE ratios have been linked to adverse health outcomes^31,32^. PC and PE in SKM-GN increased in both sexes (Fig. 2B), and with greater increase in PE, the PC:PE ratios in SKM-GN decreased progressively in both sexes (Fig. 5A, Fig S5A). The decreased PC:PE ratios correlated negatively with maximum run speed and VO_2_max changes, and positively with %fat change (Fig. 5B), suggesting lowered PC:PE ratio was associated with improved exercise performance. These data are consistent with the elevated muscle PC and PE, a lowered PC:PE ratio, and improved insulin sensitivity in athletes compared with obese and T2D subjects^33^. In heart, most PC species increased throughout ExT (Fig. 2B, D, E; clusters 5, 6), and PE species decreased before later increasing (cluster 4; Fig S5B). This gave rise to overall reduced PC:PE ratios, most evident at 8w (Fig. 5A). This was opposite to the elevated heart PC:PE ratio observed in obese, insulin resistance mice, which also showed increased number of lipid droplets in the myocardium^34^. In WAT-SC, we observed increases in PC:PE ratios in males at 4w and 8w (Fig. 5A). This may reflect phospholipid compositional changes associated with reduced adipocyte sizes with ExT, given a decreased PC:PE ratio was reported during adipocyte differentiation^35^. This was supported by the negative association of PC:PE ratio of WAT-SC with adipocyte area and positive association with adipocyte count (Fig. 5C). Moreover, male WAT-SC PC:PE ratio was negatively associated with %fat change (Fig. 5C).

Phospholipids contain high concentrations of PUFA. Phospholipid PUFA compositions in tissues, blood, or diet have been linked to disease risks in animal models^36–38^ and in clinical studies^39^. We calculated docosahexaenoic acid: arachidonic acid (DHA:ARA) ratios in PC and PE to represent phospholipid ω−3: ω−6 ratio (Fig. 5D; by taking the ratio of the sum of species with a 16:0, 16:1, 18:0, or 18:1 in addition to a 20:4 or 22:6 in PC and PE respectively). The changes in DHA:ARA ratios were largely consistent between PC and PE, and between sexes, increasing in BAT, heart, and SKM-GN, and decreasing in liver and plasma (Fig. 5D). Moreover, DHA:ARA ratios were consistently higher in female kidney, liver, heart, SKM-GN, and plasma than males (Fig. 5D). When we directly compared species abundance between sexes in SED animals, 22:6-containing PC, PE, O-PE were generally higher in females, whereas 20:4-containing species were higher in males in kidney, liver, heart, and SKM-GN (Fig. 5E).

The top-ranking lipid species that responded to ExT (ranked by p-values, any timepoint) were predominantly phospholipids, with PE O-18:1_22:5 and PE 18:0_22:5 among the top lipids increased in several tissues in females (heart, kidney, liver, lung, SKM-GN, HIPPOC; Fig S5C). Consistent with the negative enrichment of phosphatidylserines (PS) in SKM-GN at 4w in both sexes (Fig. 2B), several PS species were among the top responsive lipids at 4w in males (Fig S5C, S5D). A similar reduction was reported in the skeletal muscle of individuals with T2D after ExT^40^. In the heart, consistent with an increased DHA:ARA ratio (Fig. 5D), 22:6-containing phospholipids mostly increased, whereas 20:4-containing phospholipids decreased (Fig. 5F). Interestingly, PC 40:6, PC 18:0_22:6 and PE 18:0_22:6 were among the top responsive lipids in male heart that increased throughout ExT (Fig. 5F, S5C). Lung phospholipids are inherently enriched in saturated species by selection and remodeling during phospholipid synthesis and turnover, with di-saturated and monounsaturated PC (principally PC 16:0/16:0, PC 16:0_14:0, PC 16:0_16:1) accounting for 70% of total surfactant PC^41,42^. Our data indicate that PC 16:0/16:0 was indeed the most abundant species in the rat lung, followed by PC 32:1 and PC 16:0_18:1 (*data not shown*). With ExT, lung PC showed evident reduction in unsaturated species and an increase in the saturation level (Fig S5E), consistent with the significant increase of PC 16:0/16:0 in males at 1w and the loss of 22:6- and 20:4-containing species, along with in PE, O-PC, and PG (Fig. 5G).

### ExT effects on lipid mediators & sphingolipids

Upon phospholipase action, PUFA released from membrane-bound phospholipids can be subsequently metabolized to produce a wide variety of oxylipins and N-acylethanolamines (NAEs), simultaneously releasing the respective lysophospholipid species^43,44^. LPC are increasingly recognized as key players associated with cardiovascular and neurodegenerative diseases^45^. We observed overall increases of LPC in plasma, heart, and SKM-GN in males (Fig. 2B, D, E; cluster 5), whereas female LPC showed initial reduction in several tissues (BAT, heart, HIPPOC, kidney, liver, plasma), but increased later in plasma (Fig. 2B). Multiple LPC species, predominantly saturated and monounsaturated C15-C18 fatty acids, also showed this down-then-up temporal trend (Fig S6A).

**Figure 6:**
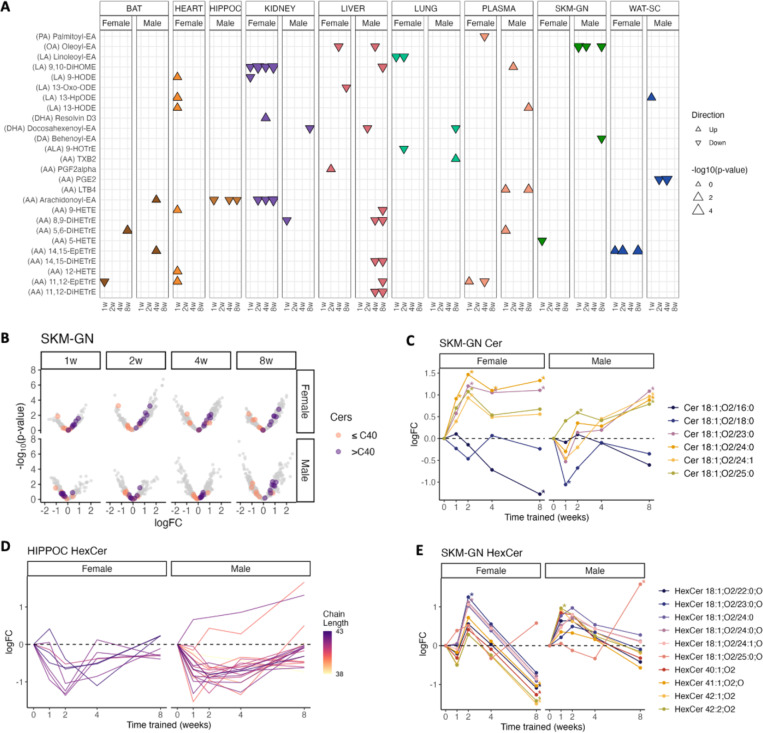
ExT effects on lipid mediators and sphingolipids (A) Differential results of targeted oxylipins and endocannabinoids that responded to exercise training at each training timepoint (p < 0.05). Direction of the triangle indicates the sign of logFC compared to sex-matched sedentary control at each training timepoint. Size of the dot indicates significance (-log_10_ p-value). (B) Volcano plots displaying comparisons of each trained group against sex-matched sedentary controls in SKM-GN. Colors highlight ceramides with ≤ or > 40 carbons (grey: all other lipids). (C) Trajectory plots of logFC of selected SKM-GN ceramides that were significantly different compared to sedentary control in each sex (* indicates p<0.05). (D) Trajectory plots of logFC of HIPPOC HexCer that were significantly different compared to sedentary control in each sex (p<0.05). Colors indicate chain length. (E) Trajectory plots of logFC of SKM-GN HexCer (* indicates p<0.05).

Oxylipins and NAEs are lipid mediators that exert diverse physiological roles in metabolic and inflammatory pathways^46,47^. We employed a targeted assay to quantify oxylipins and NAEs. Notably, males showed strong changes in eicosanoids in the liver, with significant reductions in 9-HETE, and DiHETrE and EpETrE species at later timepoints. These species are synthesized through the cytochrome P450 (CYP450) pathway from ARA (Fig. 6A, S6B). On the other hand, females showed strong responses in kidney and heart (Fig. 6A). In female kidney, FFA 20:4, FFA 22:6, LPE 18:1, LPE 20:4 and several ARA-derived oxylipins (11,12-EpETrE, 5-HETE, 8,9-EpETrE, 9-HETE), and NAEs (arachidonoyl-EA (AEA), docosahexenoyl-EA, oleoyl-EA (OEA)), showed coordinated, constant reduced levels (Fig. 6A, S6B). In female heart, several ARA- and linoleic acid (LA)-derived species showed an early spike at 1w (Fig S6B), consistent with the loss of ARA-containing phospholipids (Fig. 5F). In male lung, several ARA-derived oxylipins (5-HETE, 9-HETE, 11,12-EpETrE), LPE 16:0, and LPE 18:0 increased drastically at 8w (Fig S6B). In female lung, OEA, AEA, LPC 16:0, and LPC 18:1 showed similar increases at 8w (Fig S6B). These changes in lung oxylipin and NAEs coincided with the loss of 20:4- and 22:6- containing phospholipids (Fig. 5G), likely indicating phospholipid remodeling during pulmonary tissue adaptations that facilitates the generation of lipid mediators during ExT. Male WAT-SC exhibited drastic increases of many species at 8w, including LPC 18:1, 11,12-EpETrE, 5-HETE, 5,6-DiHETrE, 8,9-EpETrE, 9-HETE, AEA (Fig S6B), suggesting a strong inflammatory response. This is consistent with upregulation of immune pathways and immune cell proliferation in male adipose tissues, as reported in MoTrPAC Study Group, 2024^15^.

Ceramides and other sphingolipids are structural components of cellular membranes and are also important signaling molecules that influence cell cycle, cellular metabolism, and insulin sensitivity^3^. We examined changes in ceramides on the species level given their distinct functions^3^. In SKM-GN, we observed distinct ExT responses of ceramides with different fatty acyl chain lengths. Ceramides with > C40 mostly increased, whereas ceramides with ≤ C40 decreased in both sexes (Fig. 6B). Among them, Cer C16:0 and C18:0 significantly decreased, whereas Cer C23:0, C24:0, C24:1, C25:0 significantly increased (Fig. 6C). Such acyl chain length remodeling is opposite to changes associated with high-fat-diet-induced obesity^48^. Similarly in the heart, very-long-chain ceramide species (> C40) increased, driving the positive enrichment of ceramide lipid class, whereas short-chain species Cer C16:0 significantly decreased in males (Fig. 2B, S6C).

Hexosylceramides (HexCer) were highly enriched in the HIPPOC (Fig S1B) and have been implicated in the pathogenesis of neurodegenerative disorders^49^. In addition, several sphingolipid species containing nervonic acid (24:1), FFA 24:1 and SM d42:2 (likely SM d18:1/24:1) were found to accumulate with aging in mice hippocampus^49^. We observed significant negative enrichment of HexCer in HIPPOC (Fig. 2B, Fig. 6D), and reduced levels of all 24:1-containing lipids (mostly sphingolipids) during ExT, especially in males (Fig S6D). These changes in HIPPOC lipids might reflect the beneficial effects of physical exercise in preventing age-related disorders. In addition, HexCer species in the SKM-GN exhibited uniformly an initial slight increase, followed by reduction at 8w (Fig. 2B, 6E). Reduced glycosphingolipid levels (HexCer and lactosylceramide), either by inhibition of glycosphingolipid synthesis (i.e., ceramide glucosyltransferase that converts ceramides to glucosylceramides)^2^ or by calorie restricted feeding^50^, have been shown to increase insulin sensitivity in animal models. Therefore, our results might indicate improved skeletal muscle insulin sensitivity.

### Tissue connectivity and network during ExT

**Figure 7:**
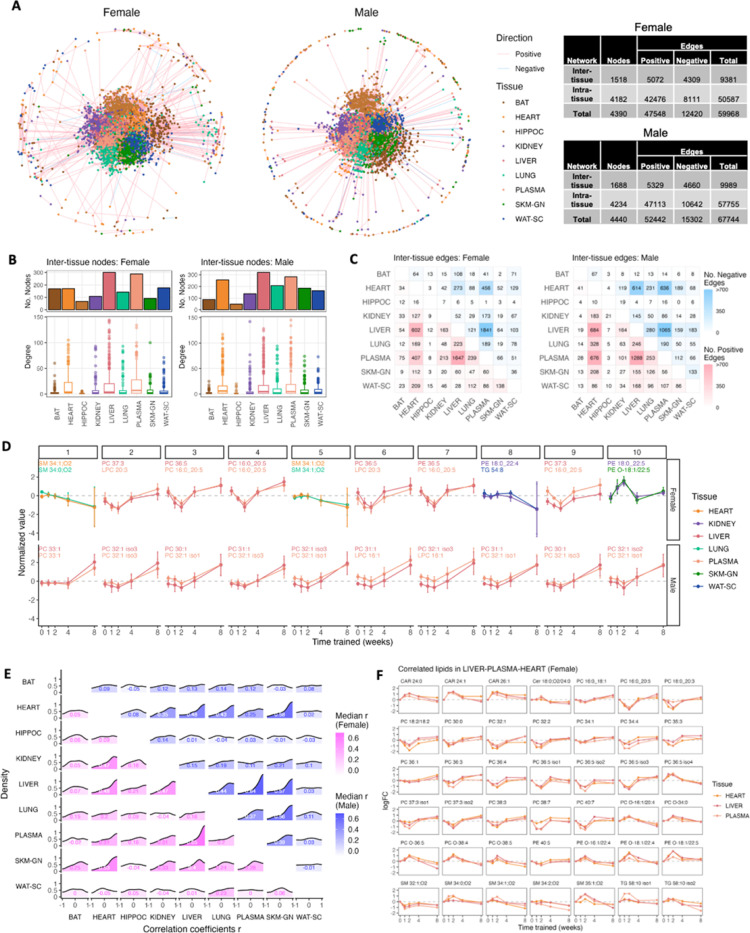
Tissue connectivity and networking during ExT (A) Networks of significantly correlated lipids in females and males (Bonferroni-adjusted p-value < 0.05). Each node indicates a lipid, and color indicates tissue. Edges indicate significant intra-tissue or inter-tissue correlations, and color indicates correlation coefficient sign (red, positive; blue, negative). Node and edge numbers are summarized in the table. (B) Number of nodes associated with each tissue in inter-tissue correlations. Boxplot of node degree (number of edges per node) of each lipid within a tissue. Box width is proportional to the number of nodes within each tissue. (C) Number of edges between each tissue pair in inter-tissue correlations. Color indicates correlation coefficient sign (red, positive; blue, negative), and the number and transparency level indicate the number of edges. (D) Trajectory plots of standardized abundance of top 10 positive inter-tissue edges (ranked by adjusted p-values). Color indicates tissue. (E) Density plot of correlation coefficients between lipid timewise logFC. Correlations were performed using logFC trajectory of the same lipid between tissue pairs for each sex. Color indicates sex, and the number and transparency level indicate the median correlation coefficients. (F) Trajectory plots of logFC of lipids that showed coordinated changes in liver, plasma, and heart in females. Lipids were selected when all three pairwise correlations were significant (p < 0.05). Color indicates tissue.

Inter-tissue communication during exercise is likely a key driver underlying the protective effects of physical activity against an array of lifestyle diseases^51^. Muscle, liver, brain, white and brown adipose tissues have all been shown to secrete proteins, peptides, and small molecules during exercise (‘exerkines’), which mediate inter-tissue communication and regulate whole-body homeostasis^51,52^. To explore tissue communication and metabolic relationships, we mapped all pair-wise metabolite correlations by constructing undirected correlation networks for each sex (Fig. 7A). A total of 4390 and 4440 nodes (i.e., lipids) and 59968 and 67744 edges (i.e., significant correlations) were detected in female and male networks, respectively. We then examined these networks by splitting into inter- and intra-tissue correlations to evaluate cross-tissue communication, and co-regulation of lipids within tissues, respectively. For inter-tissue network, plasma and liver showed the highest numbers of nodes and highest degree (i.e., number of edges per node; Fig. 7B), and the highest number of inter-tissue edges between them (Fig. 7C). Heart also showed high connectivity with plasma and liver (Fig. 7C). We hypothesize that lipids sharing positive correlations may have common origins, functions, or regulatory pathways, while negative correlations may reflect substrate flux and lipid exchange – export and uptake via circulation, and the following utilization of circulating lipids by tissues – or compensatory regulatory pathways. The top positive inter-tissue correlations (ranked by adjusted p-values) reveal coordinated changes in MUFA-containing PC and LPC between liver and plasma for males, and PUFA-containing PC and LPC for females (Fig. 7D), highlighting the active phospholipid remodeling in the liver and exchange with circulation. Overall, while there are comparable number of positive and negative inter-tissue edges, intra-tissue edges were predominantly positive (Fig. 7C, S7B). For intra-tissue correlations, the numbers of nodes within each tissue were similar (range: 427–543), with SKM-GN, WAT-SC, and plasma showing the highest degree, and lung showing the highest number of intra-tissue edges (Fig S7A, S7B), suggesting highly coordinated changes within these tissues. These analyses reveal metabolic coupling between tissues despite overall tissue-specific ExT responses.

To further identify co-regulated networks that exhibit coordinated training adaptations across tissues, we calculated Pearson correlation coefficients using the time course of logFC trajectory of shared lipids between each tissue pair (Fig. 7E). Given that plasma is the most common and accessible sample type in clinical and animal studies, we further examined the correlations between plasma and other tissues (Fig S7C). Consistent with previous results that liver and plasma showed the highest number of overlapping lipids that responded to exercise (Fig. 1D) and highest inter-tissue connectivity (Fig. 7C), they also showed the strongest coordinated training responses in both sexes (Fig. 7E). Despite both sexes displayed high liver-plasma connectivity, driven by coordinated changes in Car, PC, and O-PC, males showed higher liver-plasma correlations in Cer, DAG, and TAG than females, whereas females shower higher correlations in O-PE, and SM than males (Fig S7C). Heart also correlated well with liver and plasma (Fig. 7E, S7C). The liver-plasma-heart correlations appear to be predominantly from O-PC, PC, O-PE lipids for females (Fig. 7F, S7C). Notably, this analysis also highlighted coordinated training response between heart and SKM-GN, which was mainly driven by coordinated changes in PC and TAG (Fig S7D).

For circulating lipids, plasma ceramides better reflected liver levels for males than females (Fig S7C). Circulating DAG, commonly studied in the context of insulin resistance, best reflected male SKM-GN and liver levels but correlated poorly with all tissues for females. Plasma TAG changes correlated well with those of liver and SKM-GN (Fig S7C), potentially reflecting hepatic TAG export, mobilization, and SKM-GN uptake of TAG during exercise. Interestingly, strong negative correlations were also observed between heart and plasma acylcarnitines, kidney and plasma DAG and TAG (male only), lung and plasma TAG (female only), HIPPOC and plasma O-PE (male only) (Fig S7C). Together, our analysis underscores the role of circulating lipids in tissue communications and the central role liver plays in systemic lipid metabolism during ExT, such as providing the heart and skeletal muscle with lipids necessary to meet increasing energetic demands.

## Discussion

Here, we present the most comprehensive to-date multi-tissue lipidomic map of 8-week endurance exercise-trained male and female rats. We show profound and dynamic lipidomic remodeling after exercise that were largely tissue-, sex-, and time-dependent. We identify coordinated changes of some lipids across tissues, particularly between liver, plasma, and heart, despite highly unique tissue lipidomic composition. Many of the exercise-induced lipidomic changes we report are opposite to those observed in obesity, insulin resistance, and other cardiometabolic diseases^4,18,32^, further highlighting the systemic health effects of endurance exercise training.

### TAG utilization and remodeling during exercise training

Exercise increases energy demand, which is primarily met by accelerated carbohydrate and lipid metabolism^22^. With submaximal exercise at 70–75% VO_2_max in our study, carbohydrate utilization starts to dominate over fat oxidation^53^. It is suggested that fat oxidation at high intensity exercise is restricted by the availability of circulating fatty acids and the rate of mitochondrial fatty acid oxidation^53^. The latter was evident in our study at early training timepoints, indicated by acylcarnitine accumulation and ketone body production. Acylcarnitines are formed from their respective acyl-CoA intermediates by a family of carnitine acyltransferases to facilitate transport across the mitochondrial membranes^54^. The intramitochondrial long-chain acyl-CoAs are then oxidized and cleaved by the β-oxidation pathway. With increased fatty acid flux during exercise training, long-chain fatty acyl-CoAs could not be fully or efficiently metabolized. Acylcarnitine production has been viewed as a mechanism of mitochondrial efflux of excess acyl groups^54^. We speculate that the limitation in liver and lung FAO mainly arose from a mismatch between upregulated TCA flux and FAO flux within mitochondria. Pools of intramitochondrial long-chain fatty acyl-CoAs accumulated and drove conversion to their respective long-chain acylcarnitines, which were exported out of the mitochondria and subsequently into circulation^55^. Whereas the unique acylcarnitine profile in heart likely reflects the limitation in the endogenous supply of fatty acyl-CoAs into mitochondria given heart’s limited stores of energy substrates. Therefore, heart switches to available circulating substrates rapidly to reduce the substrate deficit^7,56^.

Ectopic lipid accumulation (spillover of lipids from adipose tissue) is associated with increased incidence of obesity and other metabolic disorders^57–59^. After 8-week endurance exercise training, male rats exhibited significantly reduced fat mass, which is consistent with drastic reduction in TAG stores in several tissues, whereas female rats maintained their body fat mass. Fatty acids derived from TAGs stored in adipose tissues are a main source of fuel during exercise and recovery, with intramuscular and intrahepatic TAGs also contributing to energy provision^60^. However, previous studies have reported inconsistent results regarding exercise training effects on intrahepatic and intramuscular TAG levels^5,8^. Here, we observed reduction in tissue total TAG content, most noticeably in the heart, liver, SKM-GN, and WAT-SC, particularly in males, confirming that endurance exercise training creates a negative lipid balance that contributes to minimizing ectopic lipid accumulation^60^. The increase in cardiac TAG content at 1w is consistent with our hypothesis that heart, at least to a certain extent, takes up circulating acylcarnitines for oxidation rather than relies on its limited endogenous energy store at early timepoints. Nevertheless, cardiac TAG content started to decrease at later timepoints, highlighting the utilization of endogenous fatty acid stores in heart with long-term exercise training^7^. The evident reduction in hepatic TAG is consistent with clinical studies that demonstrate regular exercise decreases hepatic lipid content and reduces incidence of NAFLD, which could improve whole-body insulin sensitivity and metabolic health^6,22,58^.

Our analyses also reveal the complex remodeling process within the TAG pool, that is, the relative preservation of longer-chain unsaturated TAG species at early timepoints. This has been reported in the liver and adipose tissues after acute^61^ and chronic^62^ exercises. This could be, in part, because the peroxisomal β-oxidation of polyunsaturated fatty acids is less efficient mitochondrial oxidation (generate thermal energy instead of ATP)^63^. This pattern is reversed in some sex/tissues (liver, SKM-GN, WAT-SC, male heart) at later timepoints, with a relative increase in shorter-chain and more saturated species. Along with the increased protein abundance of enzymes in DNL pathway, this suggests that DNL and re-esterification processes were likely suppressed at first (1–2 weeks), but prolonged exercise training led to the recovery of TAG synthesis. This compensatory adaptive response to fat loss was present in both sexes, but more evident in females^14^. It could be, to some extent, the result of recycling of shorter-chain fatty acids derived from incomplete oxidation (chain reduction). The accelerated TAG turnover could potentially provide more fatty acids available for oxidation^64^. It might also be a mechanism that prevents the accumulation of fatty acid metabolites and activation of inflammatory pathways that induce insulin resistance^65^.

The drastic reduction in tissue TAG stores, along with the return of acylcarnitine levels to baseline after 8-week training, suggest an accelerated but improved utilization of lipids for energy production with chronic exercise training. An increased relative contribution of fat to total energy expenditure at the same absolute intensity is one of the hallmark adaptations to exercise training^60,66^. Enhanced lipolytic rates, tissue uptake (due to increased blood flow and increased transmembrane transport), and FAO could all contribute to the elevated contribution of fat to energy expenditure during exercise^7,66^. Therefore, despite that males and females displayed different TAG utilization in response to exercise training, they both showed metabolically favorable adaptive changes that suggest improved metabolic health.

### Phospholipid remodeling during exercise training

Glycerophospholipids have been associated with obesity and cardiometabolic diseases in large-scale human cohort profiling studies^4^. Our analyses highlight the association between cardiorespiratory improvement and exercise-induced phospholipid species remodeling in metabolically active tissues. We show that the increased PC and PE, and reduced PC:PE ratio in SKM-GN were associated with improved exercise performance. These results are consistent with the effect of 12-week exercise interventions on PC, PE, and their ratios of skeletal muscle (*vastus lateralis*) in men^67^ and sedentary obese women^68^, and with comparison between endurance-trained athletes and individuals with T2D^33^. Moreover, as the lipid droplet (LD) monolayer is composed mainly of PC, followed by PE^69^, LD dynamics is influenced by PC and PE. It has been shown that PC:PE ratio decreases with LD formation^35^, whereas smaller LD size is associated with increases in PC and the PC:PE ratio; and inhibition of PC biosynthesis increases the LD size^32,69^. In male WAT-SC, the increase of PC:PE ratio, drastic reduction in TAG, and their associations with adipocyte count and area, altogether provided strong evidence that endurance training promotes favorable adipose tissue cellularity – more, smaller adipocytes that are likely metabolically healthy – and will likely limit the hypertrophic growth and hypoxia in adipose tissues^18^. In addition to PC and PE changes, we also observed reduction of PS in SKM-GN, which, similar to PE, largely resides in the inner leaflet of the plasma membrane. These might be the result of conversion between PS and PE as a part of membrane remodeling. The phospholipid remodeling in skeletal muscle, along with enhanced FAO, alludes to exercise training-induced mitochondrial adaptations that contribute, at least in part, to SKM-GN enhanced ability to oxidize lipids^5,22,30^. This may be a potential mechanism through which exercise lowers disease risks as mitochondrial dysfunction and reduction in mitochondrial mass may contribute to the dampening of skeletal muscle lipid oxidation capacity in individuals with obesity and T2D^57,60^.

With the active membrane phospholipid turnover during exercise adaptations, lysophospholipid and oxylipin abundances – both are bioactive molecules with signaling and regulatory capacities^70,71^ – showed dynamic changes in various tissues. Lung phospholipid remodeling (loss of 20:4- and 22:6-containing phospholipids) and increased production of lysophospholipids and oxylipins provide strong evidence indicative of exercise-induced pulmonary inflammation^72^ and changes in surfactant phospholipid structure. In addition, we observed drastic increases in TAG content and PC 16:0/16:0 in male lung after 1w of training. PC 16:0/16:0 is a major constituent of pulmonary surfactant phospholipids, critical for maintaining high pulmonary surfactant activity and preventing the early onset of acute lung injury^73^. One study showed that fetal rat lung fibroblasts with higher TAG content were more resistant to reactive oxygen species hydrogen peroxide, potentially because TAG were functioning as antioxidants and provided a cytoprotective effect against oxidant injury^74^. Therefore, we hypothesize that the early increases in TAG and PC 16:0/16:0 in male lung was an acute adaptative response to increased mechanical stress at the beginning of endurance training.

### Sphingolipid modifications

Ceramides are widely studied for their roles in insulin resistance and cardiovascular and metabolic diseases, with clinical profiling studies often supporting relationships between ceramides in the circulation, skeletal and cardiac muscles with adverse outcomes and increased disease risks^2,38,75^. However, studies have reported conflicting findings including both increases^76^ and reduction^40,77^ in total ceramide content in skeletal muscles after exercise. Recent discussions have highlighted different effects exerted by ceramide species with specific fatty acyl chain length^48,59^. We show the dynamic responses of individual ceramide species in SKM-GN and heart after exercise. Mainly, we observed C16:0 ceramide decreased in both SKM-GN and heart, while very-long-chain ceramides predominantly increased, consistent with a previous study that shows increased C24:0 ceramide in skeletal muscle of exercised obese women^68^. While C16:0 and C18:0 ceramides are considered central to the development of obesity, insulin resistance, and metabolic diseases^59,78^, there is insufficient evidence to support negative regulatory effects of very-long-chain ceramides on insulin sensitivity and metabolic outcomes^2^. A better understanding of individual sphingolipid species and their changes in different physiological and pathological contexts is much needed.

### Sex differences

We observed profound sex differences in tissue lipidomes at baseline. We document that nearly 70% of the plasma lipids were sexually different, and up to 55% (liver) in tissues. Moreover, sex differences in lipid abundances were largely maintained throughout the 8-week endurance training. The marked sex differences in metabolism are exemplified by a metabolomics analysis that shows one third of serum metabolites within the glucose, fatty acid, and amino acid metabolism pathways showed sex-specific abundance in healthy individuals^79^. Sexually distinct metabolism is also evident when challenged with diet- or age-induced obesity, leading to different risks to insulin resistance in rodents^80^. Here, we also observed higher abundance of DAG at baseline and enhanced reduction in male tissues (liver, BAT, plasma). This is consistent with the fact that women, with higher subcutaneous adiposity, are more sensitive to insulin^80^, and that exercise training favors greater improvement of male metabolic profile and insulin sensitivity than females^14^. Our findings also caution against using the same circulating lipid biomarkers for females and males without sex-segregated validation (e.g., ceramides). Our study included a limited sample size for sex comparisons, but we emphasize the importance for future investigations to include both sexes to better understand the sex differences in metabolism.

## Conclusions

Our study has demonstrated that eight tissues and plasma, with distinct lipidomic features, underwent dynamic, sexually dimorphic lipidomic remodeling in response to 8-week endurance exercise training. Studying both male and female rats reveal profound sexual dimorphism in lipid utilization and adaptation to exercise training. While both males and females exhibited enhanced utilization of lipid stores and improved cardiorespiratory fitness, females showed an adaptive response of preferential retention of TAGs, whereas males showed continuous and even intensified utilization of lipid stores and stronger inflammatory response with prolonged exercise training. These exercise-induced alterations in the content and composition of glycerolipids and phospholipids were associated with improved exercise performance, and reduced adiposity. The exercise-induced fatty acid remodeling, likely through fatty acid oxidation, biosynthesis, and transacylation reactions, can have important physiological implications in cellular membrane fluidity, signal transduction, and inflammatory responses. These data will improve our understanding of how exercise-mediated changes in lipid metabolism promote adaptations that improve metabolic health, and provide important information about the molecular mediators that define the metabolic responses to endurance exercise training, potentially useful for developing therapeutic strategies to prevent and mitigate metabolic diseases. Future studies, such as the ongoing MoTrPAC clinical studies, are needed to assess the translational relevance of the findings to human studies. Metabolomics/lipidomics studies using tracer techniques will also be useful in determining the impact of exercise on tissue crosstalk and metabolic flux.

## Methods

### Study design

Male and female Fischer 344 rats began endurance exercise training (ExT) at 6 months of age, following treadmill familiarization. ExT consisted of 1 hour of treadmill running at approximately 70–75% VO_2_max 5 days per week, and lasted for 1, 2, 4 or 8 weeks. A sedentary control group (SED) followed a similar schedule but was placed on the stationary treadmill for 15 min each training day for 8 weeks. All animals were placed on a reverse dark–light cycle. More detailed training protocols were described by Schenk et al.^16^. Brown adipose tissue (BAT), heart, hippocampus (HIPPOC), kidney, liver, lung, plasma, skeletal muscle gastrocnemius (SKM-GN), and subcutaneous white adipose tissue (WAT-SC) were harvested from 5 animals (per sex per group) 48 hours after the last training session. All animal procedures were approved by the Institutional Animal Care and Use Committee at the University of Iowa.

### Non-targeted lipidomics

#### Sample preparation

All solvents were LC-MS grade and purchased from ThermoFisher Scientific. All stable isotope-labeled internal standards (ISTD) were purchased from Avanti Polar Lipids (Alabaster, Alabama). The ISTD used were PC 15:0–18:1(d7); PE 15:0–18:1(d7); PS 15:0–18:1(d7); PG 15:0–18:1(d7); PI 15:0–18:1(d7); LPC 18:1(d7); LPE 18:1(d7); Chol Ester 18:1(d7); DAG 15:0–18:1(d7); TAG 15:0–18:1(d7)-15:0; SM 18:1(d9); and cholesterol (d7). ISTD were added to the extraction solvent at a final concentration in the 0.1–8 μg/ml range.

##### Plasma samples:

Samples were stored at −80 °C until extraction. An ice bath was used to thaw and maintain temperature throughout preparation. Plasma samples (25 μL) were vortex mixed with 75 μL of the extraction solvent (isopropanol containing the ISTD mix listed above) followed by centrifugation for 5 min at 21,100 x G to pellet insoluble material. The supernatant was transferred to an autosampler vial and stored at 4 °C until analysis. An aliquot from each supernatant was combined to create a pooled sample used as a quality control (QC).

##### Tissue samples:

Samples were stored at −80 °C until extraction. An ice bath was used to thaw the samples. Cryopulverized tissue samples (10 mg) with 400 μl of the extraction solvent (isopropanol containing ISTD mix listed above) were extracted by freeze-thawing in liquid nitrogen for 1 min followed by sonication in an ice bath for 3 min, repeated three times. Samples were vortex mixed for 5 min in pulsed mode followed by centrifugation for 5 min at 21,100 X G. The supernatant was transferred to an autosampler vial and stored at 4 °C until analysis. An aliquot from each supernatant was combined to create a pooled sample. Sample blanks and consortium-wide reference samples were prepared for analysis using identical methods.

#### Data acquisition

Lipid LC-MS data were acquired using a Vanquish (ThermoFisher Scientific) chromatograph fitted with a ThermoFisher Scientific Accucore^™^ C30 column (2.1 × 150 mm, 2.6 μm particle size), coupled to a high-resolution accurate mass Q-Exactive HF Orbitrap mass spectrometer (ThermoFisher Scientific) in both positive and negative ion modes. The mobile phases were 40:60 water:acetonitrile with 10 mM ammonium formate and 0.1% formic acid (mobile phase A) and 10:90 acetonitrile:isopropyl alcohol, with 10 mM ammonium formate and 0.1% formic acid (mobile phase B). The column temperature was set to 50°C, and the injection volume was 2 μL. The gradient program is shown in Table S4.

For analysis, the electrospray ion source was operated at a vaporizer temperature of 425°C, a spray voltage of 3.0 kV for positive ion mode and 2.8 kV for negative ion mode; sheath, auxiliary, and sweep gas flows of 60, 18, and 4 (arbitrary units), respectively, and capillary temperature of 275°C. The instrument acquired full MS data with 240,000 mass resolution over the 150–2000 *m/z* range. Samples were analyzed in random order with pooled QC injections collected at minimum every tenth injection.

LC-MS/MS experiments were conducted using a data dependent acquisition (DDA) strategy to aid in compound identification. MS^2^ spectra were collected with a resolution of 120,000 and the dd-MS^2^ experiments were conducted at a resolution of 30,000 with an isolation window of 0.4 *m/z* and a loop count of top 7. Stepped normalized collision energies of 10%, 30%, and 50% fragmented selected precursors in the collision cell. Dynamic exclusion was set at 7 seconds and ions with charges greater than 2 were omitted.

#### Data processing and quality control

##### Feature detection and alignment:

Peak detection, spectral alignment, and gap filling were performed with Compound Discoverer V3.0 (ThermoFisher Scientific) to yield an aligned feature table containing m/z, RT, and relative peak areas.

##### Drift correction:

Drift correction was performed on each individual feature, where a linear curve was fitted to the pooled QC sample peak areas across the batch and was then used to correct the peak area for that specific feature in the samples.

##### Annotation:

Lipid annotations were accomplished based on accurate mass and relative isotopic abundances (to assign elemental formula), retention time (to assign lipid class), and MS2 fragmentation pattern matching to local spectral databases built from curated experimental data. When possible, features were matched to authentic compounds at an MSI level 1^81^. Features that were matched to local databases were annotated to MSI level 2. Those with only compound-class annotations were assigned an MSI level 3. Unknown features were assigned MSI level 4. Lipid annotations are highly subject to the available structural information to assign alkyl chain lengths, alkyl chain position, double bond position, and double bond stereochemistry. Annotations reflect the available structural information, which sometimes results in a feature with multiple possible lipid structures. Lipid nomenclature and classification were based on the Metabolomics Workbench RefMet database (https://www.metabolomicsworkbench.org). Based on the Refmet classification, we used ‘lipid class’ throughout the analyses, which was mainly based on ‘sub class’. ‘Main class’ was adopted in lieu of ‘sub class’ when the latter consisted of <5 lipid species, except for sub class cholesterol esters (CE), in which case CE and sitosterol ester (SiE) were used to distinguish their biological origins.

##### Data cleaning and degeneracy removal:

Compound Discoverer V3.0 was used to group isotopic peaks as well as adduct ions to simplify the feature table. Some detected features were filtered from the dataset using the following criteria: (1) features with abundance lower than 5x the background signal in the sample blanks, (2) features that were not present in at least 50% of the pooled QC, (3) features with a coefficient of variance (CV) in the pooled QC higher than 30%. Redundant features (MSI levels 1–3) that were detected by both electrospray ionization (ESI) modes and/or with different adducts were filtered out by comparing the QC CV and the feature with higher CV was removed. Only annotated features (MSI levels 1–3) were included in the downstream analyses, but the complete dataset is available (see Data availability below).

##### Outlier (sample) identification:

For each dataset (tissue*ESI mode, annotated features), each sample’s correlation value was calculated against the other N-1 samples using raw peak intensity data. We found all median correlations were above the chosen threshold, 0.75. Then, principal component analyses (PCA) were performed separately for each dataset. Outliers were defined as samples outside of three times the interquartile range for at least one of the first three principal components (each explained at least 7.5% of variance in the data). No outliers were identified using these two approaches.

#### Data normalization

Raw peak intensities were log_2_-transformed. We first performed feature-wise standardization (median-centering, scaling by standard deviation). Then, we performed sample-wise normalization by median-centering samples to mitigate effects of differences in amount of starting material for each sample. Data normalization was performed separately for data acquired from positive and negative ion modes given the orders of differences in peak intensities from the two modes. Normalized data from positive and negative modes were combined for downstream analyses.

We also performed semi-absolute quantification of the annotated lipids. The concentrations of each lipid species were calculated by normalizing their peak area by that of the internal standard (ISTD) from its own class and the same ESI mode, and then multiplying by the known ISTD concentration. For lipids that did not have a matching ISTD, the ISTD with the closest chemical structure and retention time was chosen (Table S5). After calculating the relative lipid abundance, we calculated the lipid sub_class sums for each sample and identified outliers on the sample lipid class level within each tissue x ESI mode. Sample-level values that were outside of 5 median absolute deviations (MAD) of median were identified as outliers, and the entire lipid class was removed. For WAT-SC, 15 MAD was used instead. A total of 117 cases out of 16,550 were removed (case = lipid class x ESI x tissue), equivalent to 2673 out of 278,774 (0.96%) individual values. The missing values were imputed using the NIPALS algorithm from the mixOmics package^82^. The resulting negative imputed values were replaced by feature mean. ISTD-normalized data are reported as ug/mg tissue or ug/ul plasma. ISTD-normalized data were used to characterize tissue lipid class abundance profile, and to calculate ratios (PC:PE ratio, DHA:ARA ratio).

### Targeted lipidomics

#### Sample preparation

Targeted profiling of lipids was performed using an automated solid phase extraction workflow, following previously published methods^83,84^. Briefly, powdered tissue samples (10 mg) and plasma samples (100 μL) were homogenized in 100 μl PBS with a Bead Ruptor (Omni International, Kennesaw, GA). Homogenized samples were diluted with 300 μL 20% methanol and spiked with 1% BHT solution to a final BHT concentration of 0.1% and pH of 3.0 by acetic acid addition. Samples were then centrifuged (10 minutes, 14,000 rpm), and the supernatants were transferred to 96-well plates for further extraction. The supernatants were loaded to Isolute C18 SPE columns (conditioned with 1000 μL ethyl acetate and 1000 μL 5% methanol). The SPE columns were then washed with 800 μL water and 800 μL hexane.

Oxylipins were eluted with 400 μl methyl formate. Automated solid phase extraction (SPE) was conducted with a Biotage Extrahera system (Uppsala, Sweden). The eluate was dried with nitrogen and then reconstituted with 200 μL methanol prior to LC-MS analysis. Sample blanks, pooled extract samples used as QC, and consortium reference samples, were prepared for analysis using identical methods. The external standards consisted of prostaglandin E2 ethanolamide, oleoyl ethanolamide, palmitoyl ethanolamide, arachidonoyl ethanolamide, docosahexaenoyl ethanolamide, linoleoyl ethanolamide, stearoyl ethanolamide, oxy-arachidonoyl ethanolamide, 2-arachidonyl glycerol, docosatetraenoyl ethanolamide, α-linolenoyl ethanolamide, oleamide, dihomo-γ-linolenoyl ethanolamide, decosanoyl ethanolamide, 9,10 DiHOME, prostaglandin E2–1-glyceryl ester, 20-HETE, 9-HETE, 14,15 DiHET, 5(S)-HETE, 12(R)-HETE, 11(12)-DiHET, 5,6-DiHET, thromboxane B2, 12(13)-EpOME, 13 HODE, prostaglandin F2α, 14(15)-EET, 8(9)-EET, 11(12)-EET, leukotriene B4, 8(9)-DiHET, 13-OxoODE, 13(S)-HpODE, 9(S)-HpODE, 9(S)-HODE, resolvin D3, resolvin E1, resolvin D1, resolvin D2, 9(S)HOTrE, 13(S)HOTrE, 8-iso Progstaglandin F2α. All external standards were purchased from Cayman Chemical (Ann Arbor, Michigan) at final concentrations in the 0.01–20 μg/ml range.

##### Data acquisition

Targeted oxylipin and endocannabinoid data were acquired using an ExionLC/Qtrap5500 (SCIEX, Waltham, MA) LC-MS system fitted with a ThermoFisher Scientific Accucore C18 column (100 mm × 4.6, μm particle size). Data were acquired in both positive and negative ion modes using a multiple reaction monitoring (MRM) strategy. The mobile phases were water with 10 mM ammonium acetate (mobile phase A), and acetonitrile with 10 mM ammonium acetate (mobile phase B). The chromatographic method used the gradient program described in Table S6. The column temperature was set to 50°C, and the injection volume was 10 μL for negative ion mode and 2 μL for positive ion mode. The heated electrospray ionization source was operated at a vaporizer temperature of 650°C, a spray voltage of 5.5 kV for positive ion mode and 4.5 kV for negative ion mode, curtain gas, ion source gas 1 and ion source gas 2 were 20, 60 and 50, respectively. The declustering potential, entrance potential, collision energy, and collision cell exit potential were 200, 10, 40, and 10 V for negative ion mode, respectively, and 90, 10, 47 and 18 V for positive ion mode, respectively. The MRM transition list is provided in Table S7.

#### Data processing and quality control

##### Data qualification and quantitation:

Sciex OS (AB SCIEX, Version 1.6.1) was used to process the raw LC-MS targeted data for peak detection and peak area integration. Standard curves were built for each oxylipin/endocannabinoid and calibrated against external standards. All concentration points used were in the linear portion of the curve with an R^2^ value not lower than 0.9. The concentration data were expressed as ng/mg tissue or ng/100 ul plasma.

##### Data quality check:

Pool QC signals and QC retention time reproducibility were examined by calculating the Pearson correlation coefficients among the pooled QC samples. Features with concentration CV greater than 100% and of retention time CV greater than 10% in the pooled QCs were removed from the dataset.

#### Data normalization

Lipid species with more than 20% missing values were removed and the rest of missing values were imputed with a k-nearest neighbor (KNN) algorithm using the impute R package^85^. Then, log_2_ transformation, feature-wise standardization, and sample-wise normalization were performed as described above. Normalized data were used for downstream analyses unless otherwise specified.

### Differential analysis

Differential analyses were performed using the normalized (feature standardized, sample centered) data separately for males and females, for targeted and non-targeted datasets, and for each tissue. Limma with empirical Bayes variance shrinkage (*limma::eBayes*) was used to perform differential analyses^86,87^. To identify lipids that showed ExT responses, we ran a linear mean-reference model (with intercept) with *limma::lmFit* and performed F-tests (*model.matrix(~ 1+tr); tr* is a factor variable denoting the ExT group). The moderated F-statistic combines the t-statistics for all the contrasts (i.e. all ExT timepoints versus SED) into an overall test of significance. To characterize the temporal ExT responses, we ran a linear means model without an intercept and performed time-specific t-tests (*model.matrix(~0+tr)*) between contrasts of each ExT timepoint versus the sex-matched sedentary controls by running *limma::contrasts.fit*. To identify sexual dimorphism (i.e., naïve contrast between sexes at the same ExT timepoint, e.g., male SED vs. female SED), as well as sexually-dimorphic ExT responses (i.e., contrast between male vs. female temporal ExT responses at the same ExT timepoint, e.g., (male 1w – male SED) – (female 1w – female SED)), we ran a linear means model without intercept and performed t-tests (*model.matrix(~0+tr)*) contrasting sexes in each time-matched group.

### Cluster analysis

Fuzzy c-means clustering was performed using the Mfuzz R package^88^ using all named lipids. The input data were the Z-score scaling of the group (timepoint*sex) mean of the normalized data (feature standardized, sample centered). We calculated the minimum centroid distance for a range of cluster numbers using a fuzzifier m=1.5, and the optimal number (c=9) was chosen using the ‘elbow’ method. Features within the core of a cluster, with membership values (indicating how well a feature is represented by a cluster) ≥ 0.5, were used for visualization and enrichment analysis.

### Weighted gene co-expression network analysis (WGCNA)

To find modules (clusters) of highly correlated lipids, a weighted lipid co-expression network was built with the WGCNA R package^89^. Each tissue was analyzed separately and module setting parameters were modified for each tissue to ensure the same number of modules per tissue. Briefly, a tissue-specific soft threshold power was chosen for generating the assigned network by the component-wise average values for topologic overlap matrix (TOM), and lipid species were hierarchically clustered by distance measured using TOM-based dissimilarity (1-TOM). The blockwiseModules function was used to determine the lipid modules (Arguments: *TOMType=signed, deepSplit=4, corType=bicor, mergeCutHeight=0.15, pamRespectsDendro=FALSE, verbose=3*). Soft threshold power and minModuleSize for each tissue were listed in Table S8.

Pearson correlations were calculated between tissue module MEs and phenotypes separately for males and females. To simplify the correlation results for visualization, we first selected for modules that were significantly enriched with at least one lipid class, then we selected for correlations based on lipids’ known primary roles. Namely, modules that were overrepresented by TAG were correlated with adiposity measures (changes in body weight, % fat mass, % lean mass, adipocyte area and count), and hormones; modules that were overrepresented by TAG and Car were correlated with circulating energy substrates (NEFA, glycerol, glucose, total ketones); modules that were overrepresented by phospholipids and sphingolipids were correlated with cardiorespiratory fitness and insulin sensitivity markers (body weight, max run speed, VO_2_max change, insulin, HOMA-IR). The correlation network was created using the ggraph R package^90^. The full correlation results are provided in Table S3.

### Enrichment analysis

#### Lipid Set Enrichment Analysis (LSEA)

We performed Lipid Set Enrichment Analysis (LSEA), a rank-based enrichment analysis using the FGSEA procedure^91^ (details described in Many et al^14^). LSEA determines whether any a priori defined lipid sets, i.e., ‘lipid classes’ as described above, were over-represented at the extremes (top or bottom) of a ranked list. For each LSEA, input ranking metric values were from the differential analysis results at the level of individual features. For characterizing overall ExT responses, the F-score was used as the metric and used *scoreType=“pos”* given that all metrics were positive. For characterizing temporal ExT responses, and sex contrast, we used signed −log_10_-transformed p-value as the ranking metric values, where the sign indicates the direction of the log_2_ fold-change. FGSEA was run using *fgsea::fgseaMultiLevel*. The minimal size of a lipid set was set at 10. A total of 10,000 permutations were used for the estimation of enrichment p-values and to calculate normalized enrichment scores (NES).

#### Over-representation analysis (ORA)

To determine the enriched lipid classes of the c-means clusters and the WGCNA modules, ORA was performed using Fisher exact t tests (one-sided), with all named lipids as the background. For c-means clusters, ORA was performed separately for each tissue and sex, with the core lipids (membership ≥ 0.5) within each cluster analyzed as the query. For WGCNA, hub lipids (membership ≥ 0.7) within each module were analyzed as the query. Lipid classes with a minimal size of 10 lipids were tested for over-representation.

### Network analysis

Sample-level normalized data of lipids in each tissue were used for Pearson correlation. Correlation networks were analyzed with igraph^92^ and visualized using ggraph^90^.

### Statistics and data analysis

Principal components analysis (PCA) was used to compare tissue lipidomic profiles. Sample-wise median-centering was performed because feature-wise standardization removes feature-wise scales that are part of the characteristics of a tissue. Lipids that had at least 50% representation (i.e., present in ≥5 tissues) were used (a total of 505 lipids). Missing values were replaced by the minimum level of each lipid. We then ran variance component analyses using the VCA R package^93^ to estimate the amount of contribution of tissue, sex, and timepoints to the total variability. Partial least squares-discriminant analysis (PLS-DA) was performed using mixOmics^82^ to classify the lipidomic profile based on sex and training timepoints.

To examine sex- and tissue-specific training effects, we performed Pearson correlation using the training logFC of each feature (output from differential analysis by limma) between (1) males and females for each tissue, and (2) tissue pairs. A logFC=0 was added to indicate baseline, and correlations were performed for each lipid (i.e., 5-point correlation). Correlation coefficients (*r*) were calculated and displayed as density plots.

Unless otherwise noted, all analyses and plots were generated in R and using ggplot2^94^. For all statistical tests, p-values were adjusted using the Benjamini-Hochberg procedure^95^ to account for false discovery rate (FDR). Unless otherwise noted, significant results were selected at 5% FDR. Differential abundant targeted lipids were selected using raw p-value of 0.05.

## Supplementary Material

Supplement 1

## Data Availability

All data are available in the consortium public repository (https://motrpac-data.org/) and as an R package (MotrpacRatTraining6moData). Data used in the preparation of this article were obtained from the Molecular Transducers of Physical Activity Consortium (MoTrPAC) database, which is available for public access at http://motrpac-data.org and as an R package (MotrpacRatTraining6moData). Specific datasets used include metabolomics datasets (metab-u-lrppos, metab-u-lrpneg, metab-t-oxylipneg, metab-t-etamidpos), and untargeted proteomics for young adult rats endurance training.

## Key Points

- Different tissues display unique lipidomes that respond differentially to exercise training.
- Tissue-specific lipid remodeling in response to exercise training is highly sex-dependent.
- Exercise trained animals show accelerated fatty acid oxidation and phospholipid remodeling that likely associate with reduced adiposity and improved cardiorespiratory fitness.
- Coordinated changes across tissues reflect systemic tissue communication, with liver-plasma-heart connection potentially playing a key role in systemic lipid metabolism during exercise training.

## Abbreviations

ARA: Arachidonic acid
BAT: Brown adipose tissue
CV: Coefficient of variation
DAG: Diacylglycerol
DHA: Docosahexaenoic acid
DNL: de novo lipogenesis
ExT: Endurance exercise training
FAO: Fatty acid β-oxidation
FDR: False discovery rate
HEART: Heart
HexCer: Hexosylceramide
HOMA-IR: Homeostatic Model Assessment for Insulin Resistance
HIPPOC: Hippocampus
IGR: Insulin:glucagon ratio
IHTG: Intrahepatic triglyceride
IMTG: Intramuscular triglyceride
ISTD: Internal standard
KIDNEY: Kidney
LA: Linoleic acid
LC-MS/MS: Liquid chromatography-tandem mass spectrometry
LD: Lipid droplet
LIVER: Liver
LUNG: Lung
LPC: Lysophosphatidylcholine
LPE: Lysophosphatidylethanolamine
ME: Module eigenvalues
MoTrPAC: Molecular Transducers of Physical Activity Consortium
NAE: N-acylethanolamine
NAFLD: Nonalcoholic fatty liver disease
NEFA: Non-esterified fatty acid
O-PC: Ether phosphatidylcholine
O-PE: Ether phosphatidylethanolamine
ORA: Over-representation analysis
PC: Phosphatidylcholine
PCA: Principal component analysis
PE: Phosphatidylethanolamine
PLASMA: Plasma
PLS-DA: Partial least squares-discriminant analysis
PS: Phosphatidylserine
PUFA: Polyunsaturated fatty acid
SED: Sedentary group
SKM (-GN): Skeletal muscle (gastrocnemius)
SM: Sphingomyelin
T2D: Type 2 diabetes
TAG: Triacylglycerol
WAT(-SC): (Subcutaneous) white adipose tissue
WGCNA: Weighted gene co-expression network analysis
