# Supplementary material for: Endurance Exercise Training Alters Lipidomic Profiles of Plasma and Eight Tissues in Rats: a MoTrPAC study
Source: Res Sq. 2024 Nov 21:rs.3.rs-5263273. Preprint. [Version 1] doi: 10.21203/rs.3.rs-5263273/v1 (PMC11601870; doi:10.21203/rs.3.rs-5263273/v1)
Supplement: Supplement 1 [file NIHPPRS5263273V1-supplement-1.pdf]

Supplementary Files

This is a list of supplementary files associated with this preprint. Click to download.

- AuthorList.xls
- PASS1BLipidomicsSupplementarytables.xlsx
- PASS1BLipidomicssupplementaryfigureFINAL.pdf
